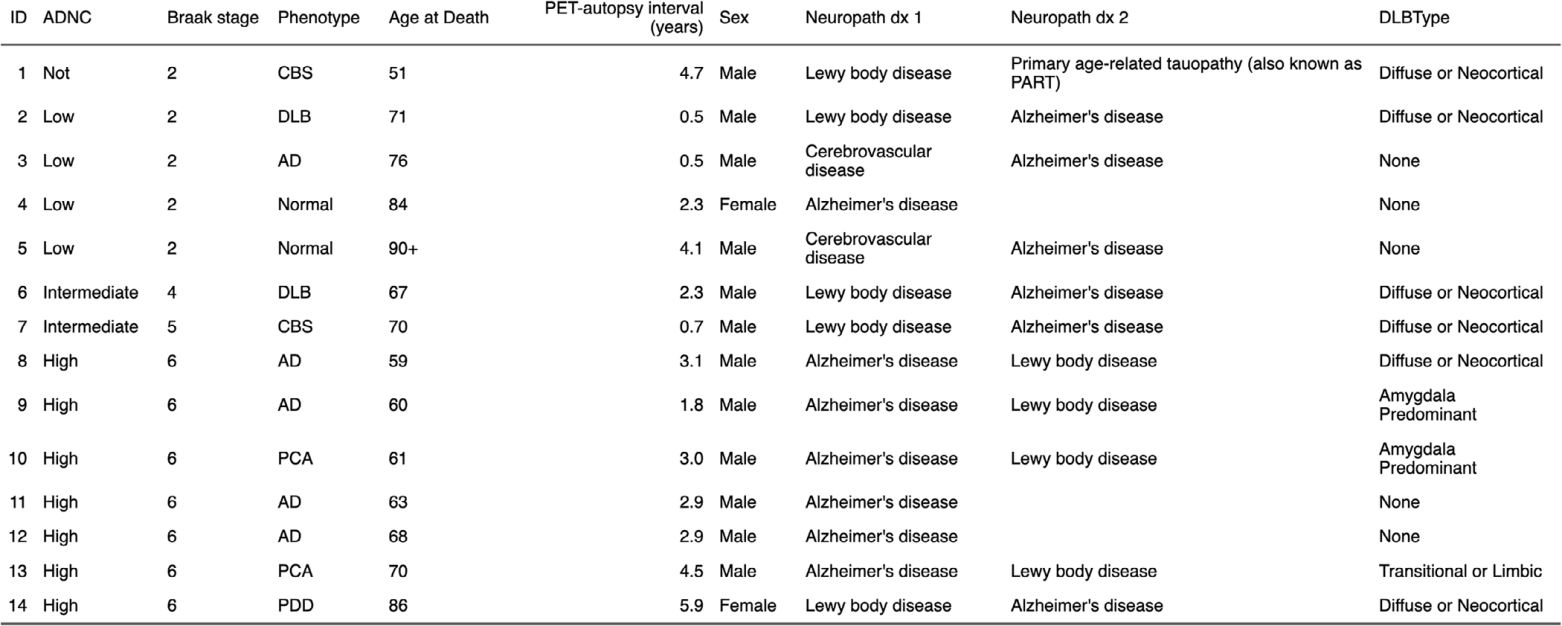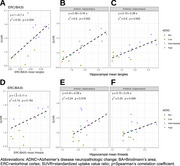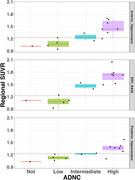# Associations of ^18^F‐flortaucipir tau PET with quantitative tau histopathology in the medial temporal lobe

**DOI:** 10.1002/alz70862_109873

**Published:** 2025-12-23

**Authors:** Jeffrey S Phillips, Amanda E Denning, Lisa M Levorse, Christopher A Brown, Laura E.M. Wisse, Sanaz Arezoumandan, Sandhitsu R. Das, Eddie B. Lee, Ricardo Insausti, John L. Robinson, Ranjit Ittyerah, Sílvia Porta, Corey T. McMillan, Daniel T Ohm, David J. Irwin, Ilya M. Nasrallah, Paul A. Yushkevich, David A. Wolk

**Affiliations:** ^1^ Penn Frontotemporal Degeneration Center, Department of Neurology, Perelman School of Medicine, University of Pennsylvania, Philadelphia, PA USA; ^2^ University of Pennsylvania, Philadelphia, PA USA; ^3^ Diagnostic Radiology, Department of Clinical Sciences Lund, Lund University, Lund Sweden; ^4^ Cleveland Clinic FL, Fort Lauderdale, FL USA; ^5^ Department of Pathology & Laboratory Medicine, Perelman School of Medicine, University of Pennsylvania, Philadelphia, PA USA; ^6^ University of Castilla‐La Mancha, Albacete Spain; ^7^ Center for Neurodegenerative Disease Research, Department of Pathology and Laboratory Medicine, University of Pennsylvania, Perelman School of Medicine, Philadelphia, PA USA; ^8^ Penn Frontotemporal Degeneration Center, Department of Neurology, Perelman School of Medicine, University of Pennsylvania, Philadelphia, PA USA; ^9^ Department of Radiology, University of Pennsylvania, Philadelphia, PA USA; ^10^ Penn Alzheimer’s Disease Research Center, University of Pennsylvania, Philadelphia, PA USA

## Abstract

**Background:**

^18^F‐Flortaucipir is widely used for positron emission tomography (PET) imaging of Alzheimer’s disease (AD)‐type tau, but its sensitivity in early Braak stages has been questioned, and hippocampal uptake is at least partially confounded by off‐target binding. We investigated associations between antemortem PET uptake and digitally‐quantified tau and TDP‐43 neuropathology.

**Methods:**

Participants (*n* = 14, Figure 1) included 5 people with no/low AD neuropathologic change (ADNC) at autopsy, 2 intermediate, and 7 high. Clinical diagnoses included normal cognition (*n* = 2), AD (*n* = 5), dementia with Lewy bodies (*n* = 2), Parkinson’s disease dementia (*n* = 1), corticobasal syndrome (*n* = 2), and posterior cortical atrophy (*n* = 2). We used the Automated Segmentation of Hippocampal Subfields T1 MRI pipeline to segment anterior and posterior hippocampus, Brodmann’s areas (BA) 35 (transentorhinal cortex) & 36, and entorhinal cortex. ^18^F‐Flortaucipir standardized uptake value ratios (SUVRs) were computed relative to inferior cerebellar grey matter and averaged across hemispheres. Postmortem sampling comprised hippocampal subiculum, CA1, CA2, CA3, and dentate gyrus; BA35 and BA36; and entorhinal cortex. FFPE‐brain tissue was immunostained using PHF1 and phospho‐specific TDP‐43 antibodies and digitally imaged. Two different weakly supervised learning algorithms, Wildcat, were trained to identify tau tangles and threads; or somatic and neuritic phosphorylated TDP‐43 (pTDP‐43) inclusions. Each pathology type was quantified by summary statistics on Wildcat heatmaps, averaged over hippocampal subfields; and over BA35/entorhinal cortex (Denning et al., 2024). We computed non‐parametric correlations between SUVRs and pathology measures at a=0.05 with false discovery rate correction.

**Results:**

Tangles were associated with SUVRs in BA35/entorhinal cortex (Spearman’s r=0.59, *p* = 0.029; Figure 2A). The mean hippocampal tangle measure was associated with SUVRs in both anterior (r=0.77, *p* = 0.002) and posterior (r=0.77, *p* = 0.002) hippocampus (Figure 2B‐C). Associations between SUVR and tau threads were marginally significant (Figure 2D‐F). In BA35/entorhinal cortex, 9/9 intermediate‐high ADNC cases had SUVRs above an established positivity cutoff of 1.23 (Figure 3). PET SUVRs were not associated with somatic or neuritic pTDP‐43 measures.

**Conclusion:**

Results suggest ^18^F‐flortaucipir is sensitive to tau burden in early Braak‐stage regions and primarily reflects neurofibrillary tangles rather than thread‐like tau inclusions.